# The lumpy skin disease virus protein LSDV003 regulates MHC-I trafficking and surface expression

**DOI:** 10.1128/mbio.02359-26

**Published:** 2026-10-22

**Authors:** Yang Yang, Lu-yao Gao, Ai-Min Guo, Huai-Jie Jia, Mi Li, Xin Wu, Hong-Bing Shu, Tian Xia

**Affiliations:** 1 State Key Laboratory of Animal Disease Control and Prevention, College of Veterinary Medicine, Lanzhou University, Lanzhou Veterinary Research Institute, Chinese Academy of Agricultural Sciences 12661, Lanzhou, China; 2 Gansu Province Research Center for Basic Disciplines of Pathogen Biology, Lanzhou, China; 3 College of Veterinary Medicine, South China Agricultural University 12526, Guangzhou, China; 4 Department of Infectious Diseases, Medical Research Institute, Taikang Center for Life and Medical Sciences, State Key Laboratory for Virology and Biosafety, Zhongnan Hospital of Wuhan University, Wuhan University 89674, Wuhan, China; Albert Einstein College of Medicine, Bronx, New York, USA

**Keywords:** lumpy skin disease virus, MHC class I, immune evasion, LSDV003, *Capripoxvirus*

## Abstract

Lumpy skin disease (LSD) is a severe transboundary disease of cattle caused by lumpy skin disease virus (LSDV), which is highly transmissible and responsible for substantial economic losses worldwide. Although cellular immune responses are critical for controlling poxvirus infections, the viral strategies used by LSDV to evade cytotoxic T-cell immunity remain poorly understood. In this study, we demonstrate that LSDV infection markedly downregulates major histocompatibility complex class I (MHC-I) surface expression in host cells. Screening of LSDV-encoded proteins identified LSDV003 as a key viral factor responsible for MHC-I downregulation. Mechanistically, LSDV003 interacts with both the MHC-I heavy chain and β2-microglobulin and mediates endoplasmic reticulum (ER) retention of MHC-I via a C-terminal HNEL motif, thereby preventing its transport to the cell surface. Mutational analyses demonstrated that disruption of the HNEL motif impaired the ability of LSDV003 to alter MHC-I localization and surface expression. Furthermore, an LSDV003-deficient virus exhibited a markedly reduced capacity to suppress MHC-I surface expression. Collectively, these findings identify LSDV003 as a viral regulator of MHC-I trafficking and reveal a mechanism by which LSDV modulates host immune recognition.

**IMPORTANCE:**

Lumpy skin disease is a highly contagious viral disease of cattle that has spread rapidly across Afro-Eurasia and causes major economic losses. However, the mechanisms used by lumpy skin disease virus to evade host immune defenses remain incompletely understood. In this study, we identify a previously unrecognized lumpy skin disease virus protein, LSDV003, that reduces the surface expression of major histocompatibility complex class I molecules in infected cells. We found that LSDV003 prevents these molecules from leaving the endoplasmic reticulum by using a C-terminal retention motif, thereby altering their normal intracellular trafficking. An LSDV003-deficient virus showed a reduced ability to suppress major histocompatibility complex class I surface expression during infection. These findings reveal an important immune evasion strategy used by lumpy skin disease virus and highlight LSDV003 as a potential target for improved vaccines and antiviral approaches to control this economically important disease.

## INTRODUCTION

Lumpy skin disease virus (LSDV) belongs to the genus *Capripoxvirus* of the family *Poxviridae* (1). LSDV primarily infects cattle and water buffalo, causing lumpy skin disease (LSD) (2, 3). Traditionally confined to Africa and the Middle East, this vector-borne disease has rapidly spread across Europe and Asia in recent years due to climate change and increased economic activity (4). Although the mortality rate of LSD is generally low, the disease can lead to chronic debilitation, reduced milk production, abortion, infertility, hide damage, and even death. As a result, LSD significantly impacts animal health and welfare (5, 6). Due to its high morbidity and substantial economic burden on the livestock industry, LSD is listed as a notifiable disease by the World Organization for Animal Health.

LSDV infection triggers both innate and adaptive immune responses in the host (7). The innate immune system serves as the first line of defense, followed by the adaptive immune response, which provides highly specific and long-lasting protection (8). Adaptive immunity consists of both humoral (antibody-mediated) and cellular (cell-mediated immunity, CMI) responses, which work together to neutralize free virus particles and eliminate infected cells. Cellular immunity, which involves the activation of antigen-specific T lymphocytes and other effector cells, plays a critical role in the clearance of intracellular pathogens (9). However, the role of cellular immunity in LSDV infection remains poorly understood. Elucidating its function could provide key insights into the immunopathogenesis of LSD and guide the development of antiviral drugs and vaccines.

Cytotoxic T lymphocytes (CTLs) play a central role in controlling viral infections by directly eliminating infected cells. They recognize viral antigens presented by major histocompatibility complex class I (MHC-I) molecules on professional antigen-presenting cells (APCs) during priming and on infected nucleated cells during effector responses (10). MHC-I consists of a transmembrane heavy chain non-covalently associated with β2-microglobulin (β2m) (11). The MHC system is referred to as the human leukocyte antigen (HLA) system in humans and the bovine leukocyte antigen (BoLA) system in cattle (12, 13). During viral replication, viral proteins are degraded by the proteasomes in the cytosol into peptides, which are then transported into the endoplasmic reticulum (ER) by the transporter associated with antigen processing (TAP). In the ER, newly synthesized MHC-I heavy chains and β2m associate with the peptide-loading complex, which includes TAP, tapasin, ERp57, and calreticulin. Peptide loading stabilizes the MHC-I complex, allowing mature peptide–MHC-I complexes to exit the ER and traffic to the plasma membrane (14).

To evade CD8^+^ T-cell-mediated recognition, many viruses have evolved strategies to interfere with the MHC-I antigen presentation pathway. These include inhibition of proteasomal degradation, suppression of TAP or tapasin function, retention of MHC-I molecules within the ER, and enhanced internalization and degradation of MHC-I from the surface of APCs (15). For example, Kaposi’s sarcoma-associated herpesvirus encodes two E3 ubiquitin ligases, K3 and K5, which promote the internalization and degradation of MHC-I molecules (16, 17). The human-specific poxvirus molluscum contagiosum virus encodes MC80, which interferes with MHC-I antigen presentation by targeting peptide-loading components for ER-associated degradation, resulting in ER retention of MHC-I and viral immune evasion (18). Cowpox virus (CPXV) encodes CPXV012 and CPXV203, which inhibit TAP-mediated peptide transport and sequester MHC-I molecules within the ER through a C-terminal KTEL motif, respectively (19–21). Similarly, SARS-CoV-2 accessory proteins ORF7a and ORF3a reduce surface MHC-I expression through distinct post-translational mechanisms, and ORF8 targets MHC-I for autophagy-mediated degradation, collectively impairing antigen presentation (22, 23). However, whether LSDV has evolved similar mechanisms to interfere with antigen presentation via MHC-I is unknown.

In this study, we demonstrate that LSDV infection markedly downregulates host MHC-I surface expression. Screening of LSDV-encoded proteins identified LSDV003 as a potent negative regulator of MHC-I expression on the surface of both human and bovine cells. Mechanistically, LSDV003 interacts with MHC-I molecules in the ER and prevents their trafficking to the cell surface via its C-terminal HNEL motif. Notably, deletion of LSDV003 abolished the ability of LSDV to suppress MHC-I surface expression. Consistent with these findings, cells infected with the LSDV003-deficient virus induced enhanced CD8^+^ T-cell activation in splenocytes from LSDV-infected mice. Collectively, these results identify LSDV003 as a key viral immune evasion factor that enables LSDV to modulate MHC-I surface expression and impair antiviral cellular immune responses.

## RESULTS

### LSDV infection downregulates cell surface expression of MHC-I molecules

**Fig 1:**
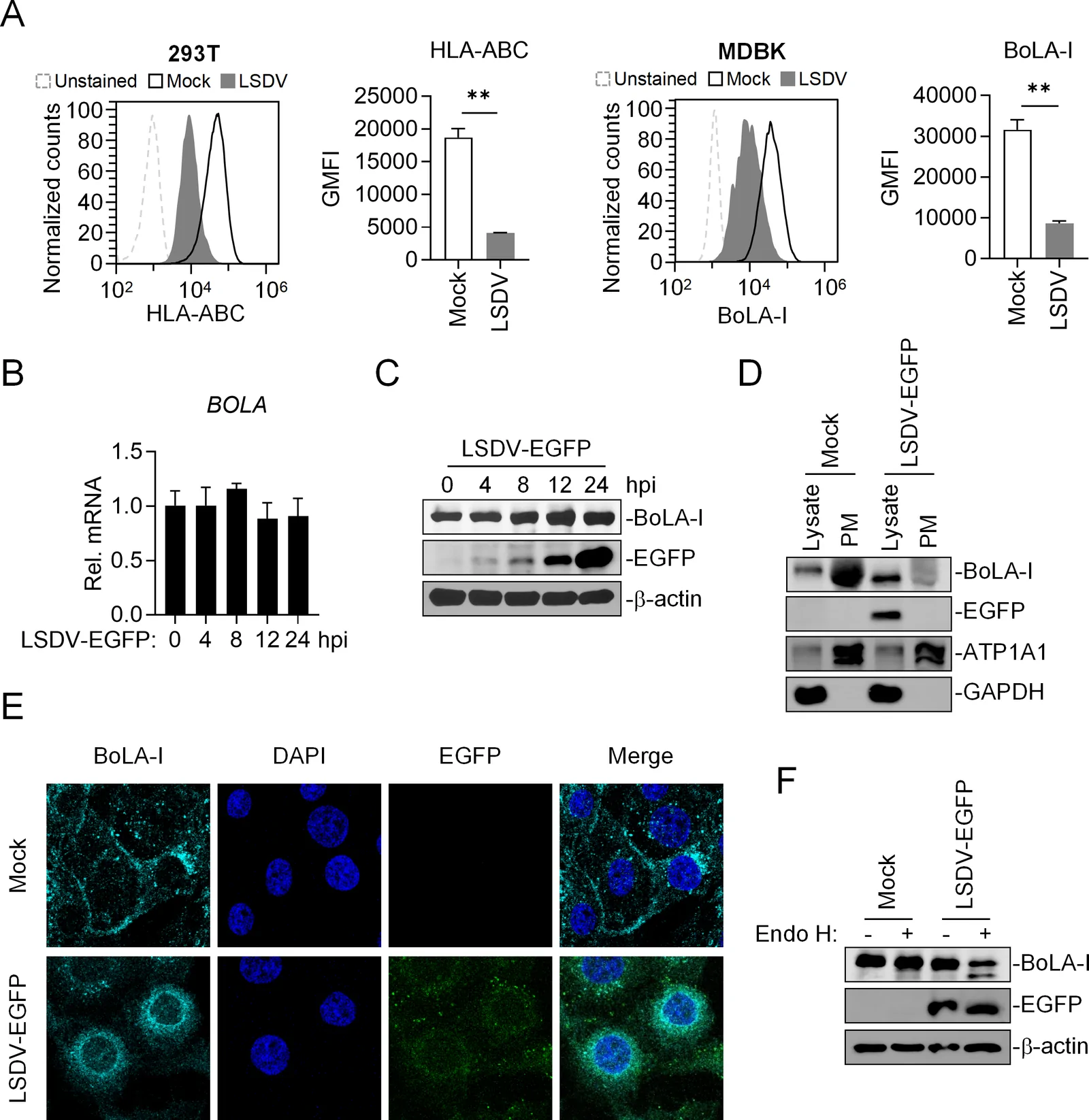
LSDV infection reduces MHC-I surface expression. (**A**) Flow cytometric analysis of MHC-I surface expression following LSDV infection. HEK293T and MDBK cells (5 × 10^5^) were infected with LSDV-EGFP (MOI = 1) for 24 h, and MHC-I surface expression was assessed using flow cytometry. Data are presented as mean ± SD (*n* = 3). **, *P* < 0.01 (unpaired *t*-test). (**B**) Effects of LSDV infection on MHC-I mRNA levels. MDBK cells (5 × 10^5^) were infected with LSDV-EGFP (MOI = 1). Total RNA was collected at the indicated times and analyzed by RT-qPCR. (**C**) Effects of LSDV infection on total MHC-I protein levels. MDBK cells (5 × 10^5^) were infected with LSDV-EGFP (MOI = 1), harvested at the indicated times, and analyzed by immunoblotting. (**D**) LSDV infection reduces plasma membrane MHC-I. MDBK cells (3 × 10^7^) were infected with LSDV-EGFP (MOI = 4) for 24 h. Surface proteins were biotinylated, isolated using streptavidin beads, and analyzed by immunoblotting. (**E**) Subcellular localization of MHC-I during LSDV infection. MDBK cells (1 × 10^5^) were infected with LSDV-EGFP (MOI = 1) for 24 h and analyzed by confocal microscopy. (**F**) Endo H sensitivity of MHC-I following LSDV infection. MDBK cells (1.5 × 10^6^) were infected with LSDV-EGFP (MOI = 4) for 24 h. Lysates were treated with Endo H for 2 h before immunoblot analysis.

To determine whether LSDV infection modulates MHC-I-mediated antigen presentation, we assessed the surface expression of MHC-I molecules in both human embryonic kidney HEK293T cells and bovine Madin-Darby bovine kidney (MDBK) cells following infection with LSDV at a defined multiplicity of infection (MOI) for 24 h. Flow cytometry analysis revealed a significant reduction in MHC-I surface expression upon LSDV infection (Fig. 1A). In contrast, RT-qPCR analysis showed that *BOLA* mRNA levels remained unchanged up to 24 h post-infection (Fig. 1B). Interestingly, immunoblotting analysis revealed a modest increase in total BoLA-I protein levels after LSDV infection (Fig. 1C). These results suggest that the reduction in MHC-I expression occurs specifically at the cell surface rather than being caused by reduced MHC-I transcription or total protein abundance.

To validate this observation, we performed cell surface biotinylation to isolate plasma membrane proteins. Immunoblotting analysis showed that while BoLA-I protein levels in whole-cell lysates were comparable between groups, plasma membrane-associated BoLA-I was markedly reduced in LSDV-infected cells relative to mock-infected controls (Fig. 1D). In contrast, the surface expression of the plasma membrane protein ATP1A1 remained unchanged. These results suggest that LSDV infection interferes with the trafficking of BoLA-I to the cell surface.

Next, we examined whether LSDV infection alters the subcellular distribution of BoLA-I. Confocal microscopy showed that in uninfected cells, BoLA-I was predominantly localized at the plasma membrane, whereas in LSDV-infected cells, it accumulated in the perinuclear region, with markedly reduced plasma membrane staining (Fig. 1E). Under normal conditions, newly synthesized MHC-I heavy chains undergo N-linked glycosylation in the ER and Golgi apparatus (24). During maturation in the Golgi, high-mannose and hybrid N-glycans are converted into complex forms that become resistant to Endoglycosidase H (Endo H) digestion (25). To determine whether LSDV infection impairs MHC-I trafficking to the cell surface, we treated cell lysates with Endo H. In mock-infected cells, most BoLA-I molecules were Endo H-resistant, consistent with normal glycan maturation. In contrast, BoLA-I in LSDV-infected cells remained Endo H-sensitive and migrated at a lower molecular weight (Fig. 1F), indicating a blockade in cis- to trans-Golgi transport. Collectively, these findings demonstrate that LSDV infection disrupts the intracellular trafficking of MHC-I molecules, preventing their transit through the Golgi apparatus and thereby reducing surface expression.

### LSDV003 downregulates cell surface expression of MHC-I molecules

**Fig 2:**
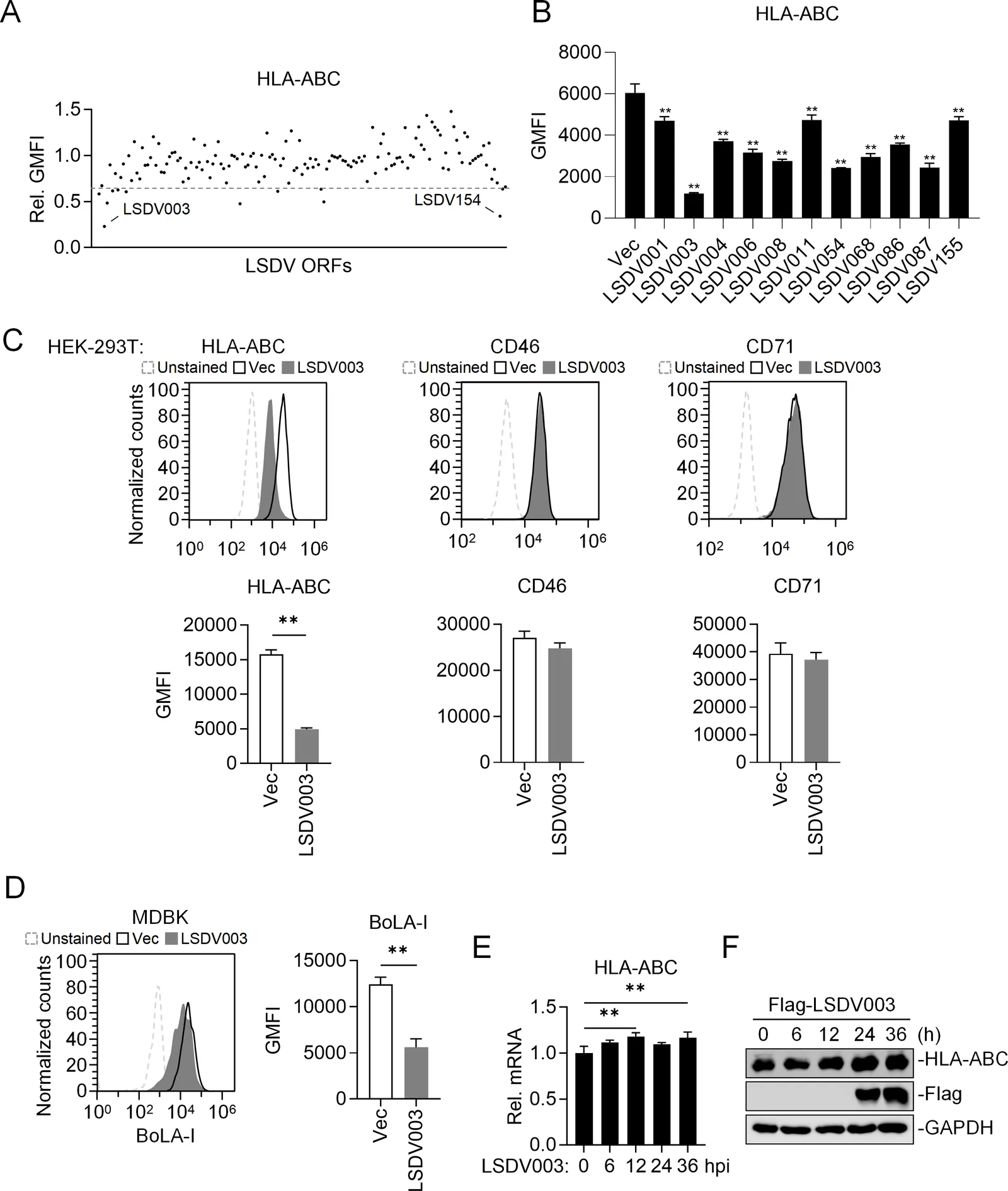
LSDV003 downregulates MHC-I surface expression. (**A**) Single-pass primary screening of LSDV proteins that downregulate MHC-I surface expression. HEK293T cells (1 × 10^5^) were transfected with individual LSDV expression plasmids (400 ng) for 24 h, and MHC-I surface expression was analyzed by flow cytometry. (**B**) Secondary validation of candidate LSDV proteins identified in the primary screen. HEK293T cells (1 × 10^5^) were transfected with the indicated LSDV expression plasmids (400 ng) for 24 h, and MHC-I surface expression was analyzed by flow cytometry. (**C**) Effects of LSDV003 overexpression on surface expression of HLA-ABC, CD46, and CD71. HEK293T cells (1 × 10^5^) were transfected with LSDV003 (400 ng) for 24 h, and surface expression of the indicated proteins was analyzed by flow cytometry. (**D**) Effects of LSDV003 overexpression on BoLA-I surface expression. MDBK cells (1 × 10^5^) were transfected with LSDV003 (400 ng) for 24 h, and BoLA-I surface expression was analyzed by flow cytometry. (**E**) Effects of LSDV003 overexpression on MHC-I mRNA levels. HEK293T cells (5 × 10^5^) were transfected with LSDV003 (400 ng). Total RNA was collected at the indicated time points and analyzed by RT-qPCR. (**F**) Effects of LSDV003 overexpression on MHC-I protein levels. HEK293T cells (5 × 10^5^) were transfected with LSDV003 (400 ng). Cells were harvested at the indicated time points and analyzed by immunoblotting. Data in panels **B–E** are presented as mean ± SD (*n* = 3). Statistical significance was determined by unpaired *t*-test (**C** and **D**) or one-way ANOVA (**B** and **E**). *, *P* < 0.05; **, *P* < 0.01.

To identify the viral proteins responsible for MHC-I downregulation, we transfected HEK293T cells with expression plasmids for 151 LSDV-encoded proteins and analyzed surface MHC-I expression by flow cytometry. The primary screen identified 12 ORFs corresponding to 11 unique candidate proteins that reduced MHC-I surface expression to less than two-thirds of that observed in vector-transfected cells (Fig. 2A). To validate these initial hits, we performed a secondary screen of the 11 candidates. This analysis identified LSDV003 as the most prominent and reproducible inhibitor of MHC-I surface expression (Fig. 2B). Notably, *LSDV003* and *LSDV154* are duplicated genes located within the inverted terminal repeat (ITR) regions of the LSDV genome and encode identical proteins. We therefore focused subsequent analyses on *LSDV003* as a representative of this duplicated locus. Ectopic expression of LSDV003 reduced surface expression of MHC-I but not the other examined surface proteins, including CD46 and CD71, in HEK293T cells (Fig. 2C). In similar experiments, ectopic expression of LSDV003 also significantly reduced the surface expression of BoLA-I in MDBK cells (Fig. 2D). RT-qPCR and immunoblotting analysis showed that ectopic expression of LSDV003 did not suppress MHC-I mRNA (Fig. 2E) or total protein (Fig. 2F) levels in HEK293T cells. These results suggest that ectopic expression of LSDV003 specifically impairs the surface expression of MHC-I in both human and bovine cells at a post-translational level.

### LSDV003 disrupts MHC-I trafficking via its HNEL motif

**Fig 3:**
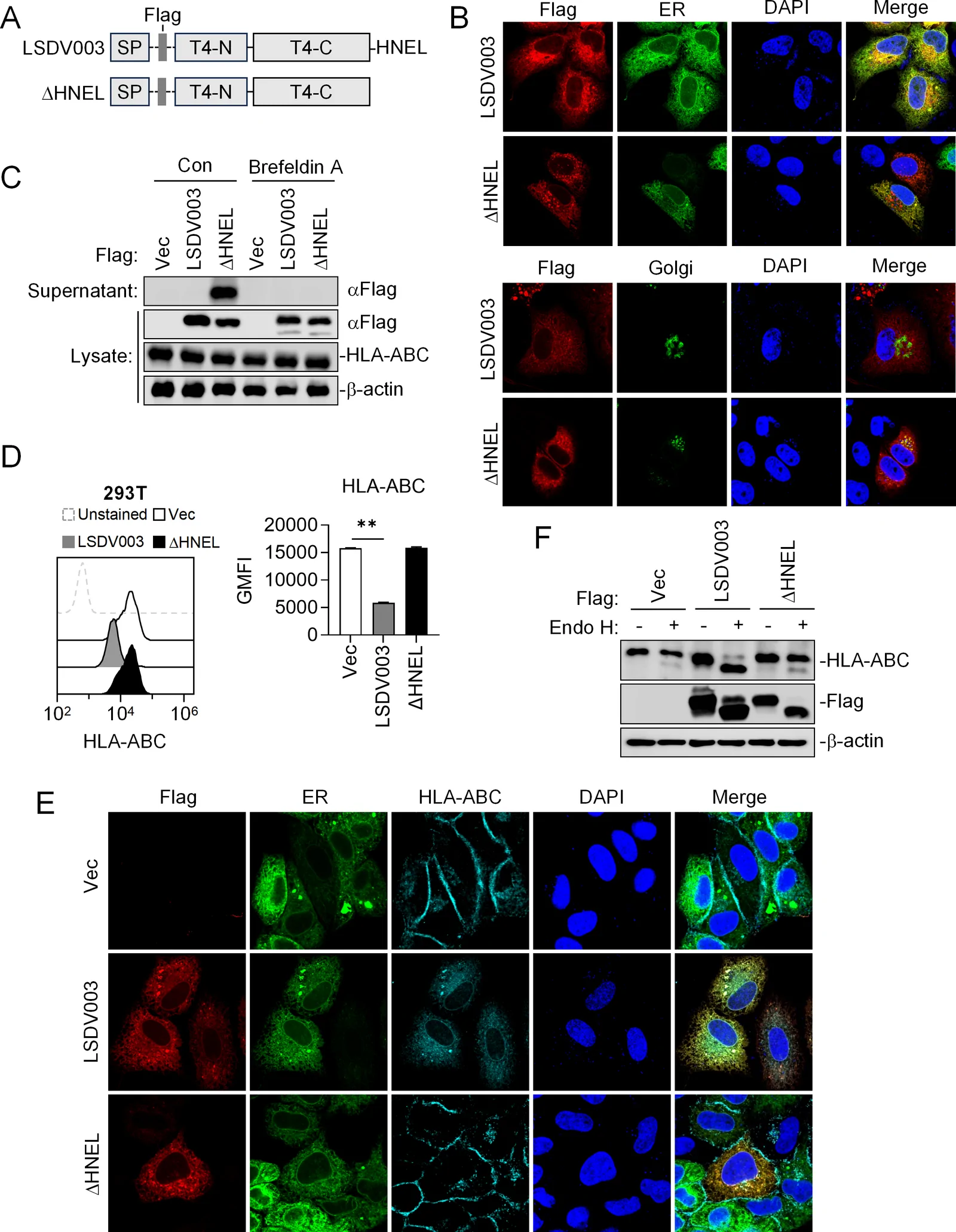
LSDV003 disrupts MHC-I trafficking via its HNEL motif. (**A**) Construction of full-length LSDV003 and HNEL deletion mutant (ΔHNEL). A Flag tag was inserted downstream of the signal peptide. (**B**) Subcellular localization of LSDV003 and ΔHNEL. HeLa cells (1 × 10^5^) were transfected with Flag-LSDV003 (200 ng) or ΔHNEL (200 ng), together with ER marker EGFP-KDEL (100 ng) or Golgi marker GM130-EGFP (100 ng), for 24 h before immunostaining and confocal microscopy. (**C**) Expression of LSDV003 and ΔHNEL in culture medium. Cells were transfected with Flag-tagged LSDV003 (400 ng) or ΔHNEL mutant (400 ng) for 24 h, followed by treatment with brefeldin A (3 μg/mL) for an additional 24 h. Cell lysates and culture supernatants were collected and analyzed by immunoblotting with the indicated antibodies. (**D**) Effects of LSDV003 and ΔHNEL overexpression on MHC-I surface expression. HEK293T cells (1 × 10^5^) were transfected with Flag-LSDV003 (400 ng) or ΔHNEL (400 ng) for 24 h. MHC-I surface expression was analyzed by flow cytometry. Data are presented as mean ± SD (*n* = 3). **, *P* < 0.01 (unpaired *t*-test). (**E**) Subcellular localization of MHC-I following LSDV003 or ΔHNEL overexpression. HeLa cells (1 × 10^5^) were transfected with an empty vector, Flag-LSDV003 (200 ng) or ΔHNEL (200 ng), together with EGFP-KDEL (100 ng), for 24 h before immunostaining and confocal microscopy. (**F**) Endo H sensitivity of MHC-I following LSDV003 or ΔHNEL overexpression. HEK293 cells (1 × 10^5^) were transfected with Flag-LSDV003 (400 ng) or ΔHNEL (400 ng) for 24 h. Cell lysates were treated with Endo H for 2 h before immunoblot analysis.

LSDV003 contains an N-terminal signal peptide, followed by a poxvirus T4 protein N-terminal domain and a C-terminal domain. Notably, its C-terminal tail harbors an HNEL motif, a putative KDEL-like ER retrieval/retention motif (26). To determine whether the HNEL motif is critical for the subcellular localization and function of LSDV003, we generated an LSDV003 mutant lacking the HNEL sequence (ΔHNEL) (Fig. 3A). Confocal microscopy revealed that full-length LSDV003 predominantly localized to the ER, whereas the ΔHNEL mutant showed additional localization to the Golgi apparatus (Fig. 3B). This shift in localization is consistent with loss of ER retention, resulting in trafficking of the protein into the conventional secretory pathway. To further assess this, immunoblotting analysis revealed that full-length LSDV003 was detected exclusively in cell lysates, whereas the ΔHNEL mutant was present in both cell lysates and culture supernatants (Fig. 3C). Importantly, treatment with brefeldin A, an inhibitor of ER-to-Golgi transport, abolished the secretion of the ΔHNEL mutant into the culture supernatant (Fig. 3C), indicating that loss of the HNEL motif permits LSDV003 trafficking through the conventional secretory pathway. Flow cytometry analysis further demonstrated that overexpression of the ΔHNEL mutant failed to inhibit the surface expression of HLA-I (Fig. 3D). These findings indicate that the HNEL motif is required to retain LSDV003 in the ER and that ER localization is critical for its ability to modulate MHC-I surface expression.

We next examined whether LSDV003 alters the subcellular distribution of MHC-I. In cells transfected with either empty vector or the ΔHNEL mutant, HLA-I was primarily localized to the plasma membrane. In contrast, cells expressing full-length LSDV003 exhibited altered subcellular distribution of HLA-I, with increased co-localization with ER markers (Fig. 3E). Consistent with these observations, Endo H digestion assays demonstrated that LSDV003, but not the ΔHNEL mutant, impaired HLA-I trafficking to the medial Golgi (Fig. 3F). Together, these results suggest that LSDV003 disrupts MHC-I trafficking in an HNEL motif-dependent manner, resulting in ER retention and impaired surface expression.

### LSDV003 interacts with MHC-I molecules

CPXV protein CPXV203, an ortholog of LSDV003 with 33.5% sequence identity based on full-length amino acid sequence alignment, has been reported to retain MHC-I molecules in the ER via its C-terminal KTEL motif (19, 27). Both CPXV203 and LSDV003 belong to the poxvirus immune evasion (PIE) domain family and exhibit similar predicted tertiary structures (28). The structure of the CPXV203–MHC-I complex has previously been resolved, showing that CPXV203 binds beneath the MHC-I peptide-binding platform and contacts both the MHC-I heavy chain (HC) and β2m (21).

**Fig 4:**
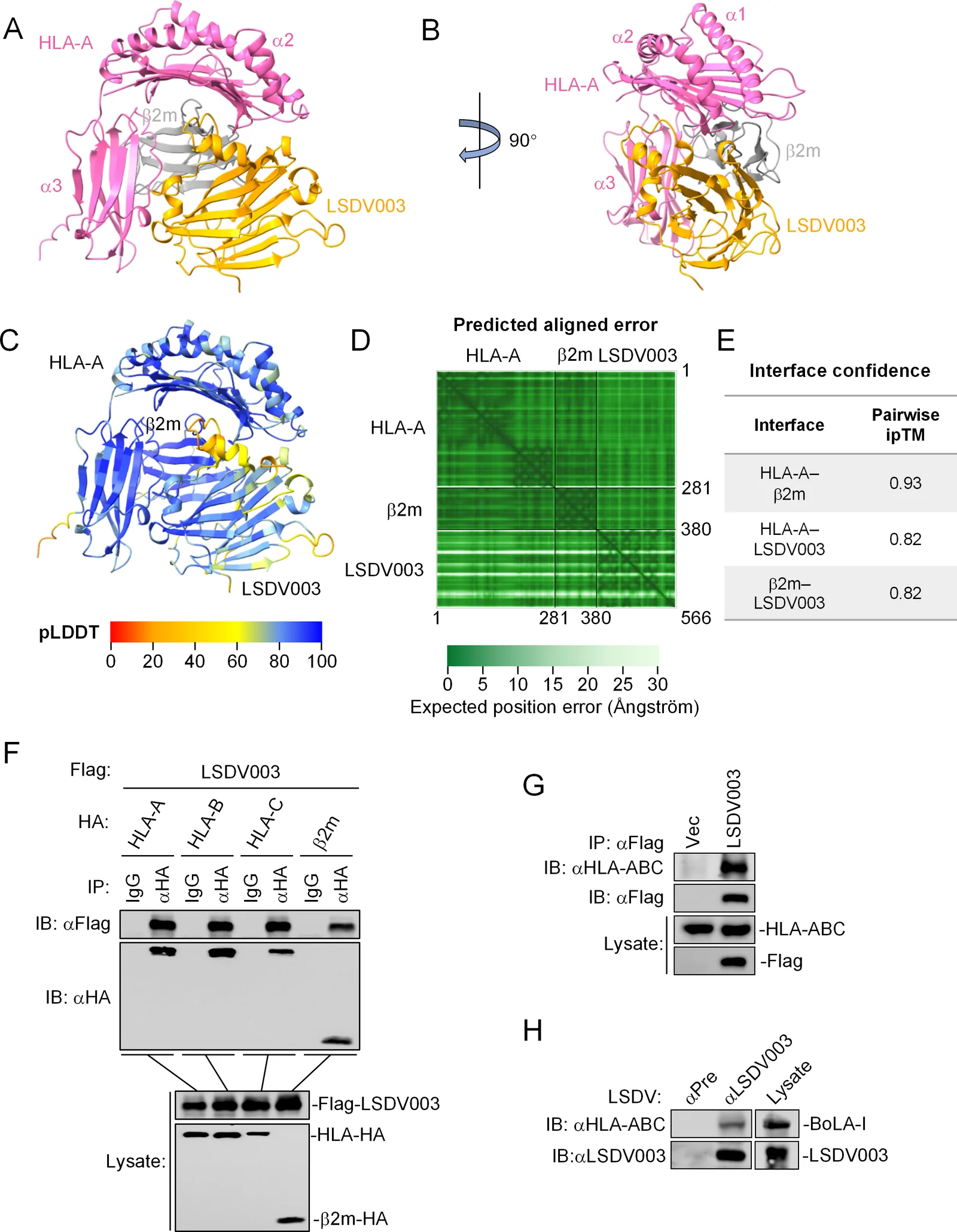
LSDV003 associates with MHC-I molecules. (**A** and **B**) Cartoon representations of the top-ranked model shown in front (**A**) and 90°-rotated (**B**) views. HLA-A, β2m, and LSDV003 are colored pink, gray, and orange, respectively. The α1, α2, and α3 domains of HLA-A are indicated. (**C**) The top-ranked model is colored according to pLDDT scores, with blue indicating higher local confidence. (**D**) PAE map of the HLA-A–β2m–LSDV003 complex with chain boundaries indicated. Darker green represents lower predicted positional error. Values are expressed in ångströms (Å). (**E**) Pairwise ipTM scores for HLA-A–β2m (0.93), HLA-A–LSDV003 (0.82), and β2m–LSDV003 (0.82). (**F**) Interaction of LSDV003 with MHC-I components. HEK293T cells (1 × 10^7^) were transfected with the indicated plasmids for 24 h, followed by co-immunoprecipitation with anti-HA antibody. (**G**) Interaction of LSDV003 with endogenous MHC-I. HEK293 cells (1 × 10^7^) were transfected with Flag-LSDV003 for 24 h, followed by immunoprecipitation with anti-Flag antibody and immunoblot analysis. (**H**) Interaction of LSDV003 with MHC-I during viral infection. MDBK cells were infected with LSDV-EGFP (MOI = 4) for 24 h, followed by immunoprecipitation with anti-serum from LSDV003-immunized mice.

Based on these observations, we hypothesized that LSDV003 may similarly interact with MHC-I molecules and thereby contribute to their retention in the ER. To explore the potential structural basis of this interaction, we used AlphaFold Server to model the HLA-A–β2m–LSDV003 complex (29). The top-ranked model reproduced the established HLA-A–β2m architecture and positioned LSDV003 along the lower surface of the MHC-I complex, where it contacted the α2 and α3 domains of the HLA-A heavy chain, as well as β2m (Fig. 4A and B). Predicted local distance difference test (pLDDT) mapping showed high local confidence for the HLA-A–β2m scaffold and most regions of LSDV003 (Fig. 4C). Consistently, the predicted aligned error (PAE) map supported a coherent arrangement of the three subunits (Fig. 4D), with pairwise interface-predicted TM (ipTM) scores of 0.93 for HLA-A–β2m and 0.82 for both HLA-A–LSDV003 and β2m–LSDV003 (Fig. 4E). Together, these analyses predicted a plausible interaction interface between LSDV003 and the MHC-I complex.

To experimentally assess this prediction, we co-transfected LSDV003 with individual HLA class I heavy chains (HLA-A, -B, -C) or β2m. Co-immunoprecipitation (co-IP) assays showed that LSDV003 associated with each of the HLA class I heavy chains, as well as β2m (Fig. 4F). In addition, ectopically expressed LSDV003 co-precipitated with endogenous HLA (Fig. 4G). To determine whether this association also occurs during LSDV infection, MDBK cells were infected with LSDV and subjected to co-IP analysis. LSDV003 co-precipitated with endogenous BoLA-I during infection (Fig. 4H), further supporting an association between LSDV003 and MHC-I molecules under physiologically relevant conditions.

### R50 contributes to the LSDV003–MHC-I interaction

**Fig 5:**
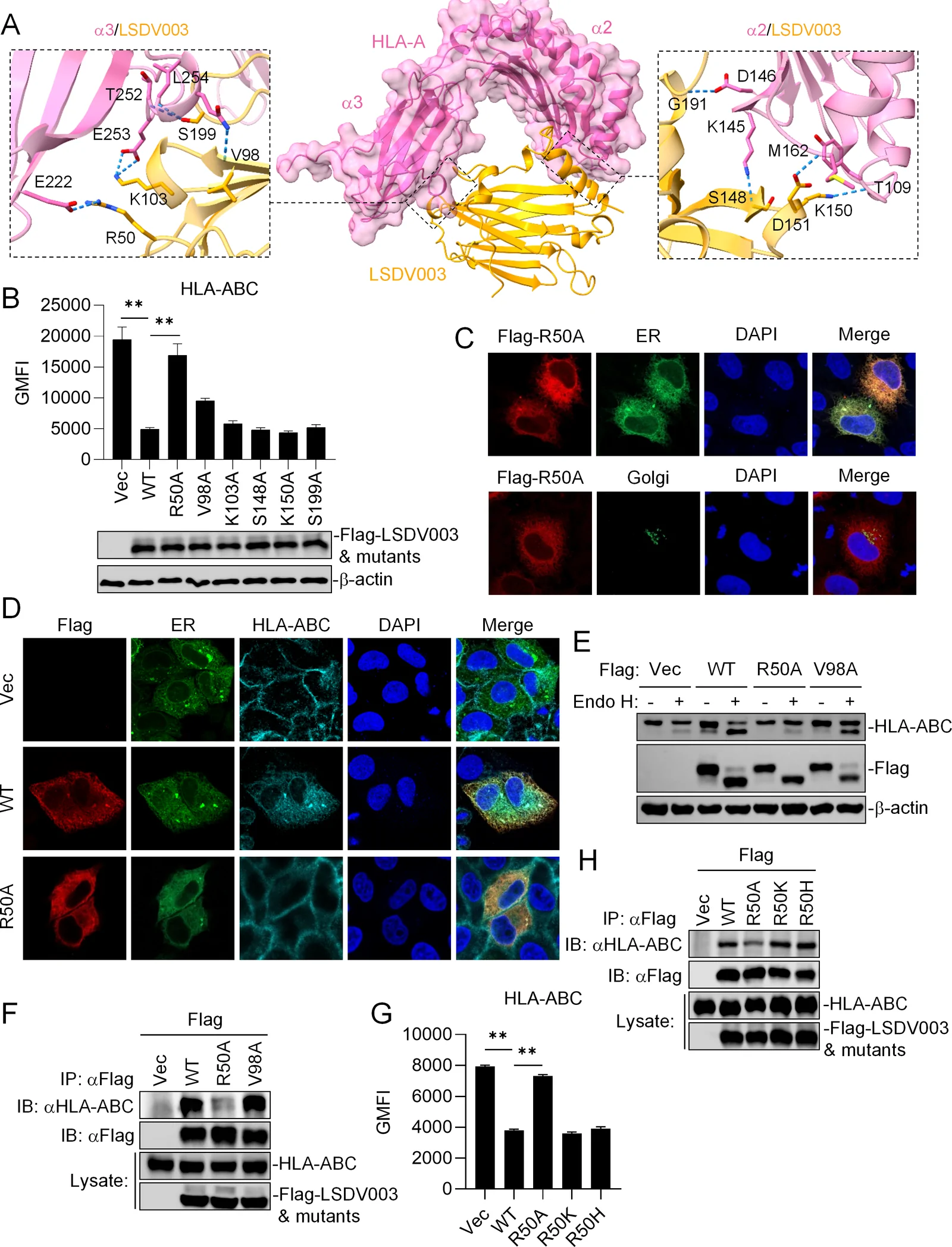
R50 contributes to the LSDV003–MHC-I interaction. (**A**) Predicted interaction interfaces between LSDV003 and HLA-A. The central image shows the top-ranked model, with HLA-A in pink and LSDV003 in orange. Enlarged views show the two contact regions adjacent to the HLA-A α3 domain (left) and α2 domain (right). Selected interface residues are shown as sticks and labeled; atom colors distinguish their chemical elements. Dashed lines indicate putative polar or electrostatic contacts inferred from the predicted structure. (**B**) Effects of LSDV003 mutants on MHC-I surface expression. HEK293T cells (1 × 10^5^) were transfected with Flag-LSDV003 or its mutants (400 ng) for 24 h. MHC-I surface expression was analyzed by flow cytometry. (**C**) Subcellular localization of the LSDV003 R50A mutant. HeLa cells (1 × 10^5^) were transfected with Flag-R50A (200 ng) together with the ER marker EGFP-KDEL (100 ng) or the Golgi marker GM130-EGFP (100 ng) for 24 h, followed by immunostaining and confocal microscopy. (**D**) Subcellular localization of MHC-I following expression of LSDV003 or the R50A mutant. HeLa cells (1 × 10^5^) were transfected with empty vector, Flag-LSDV003 (200 ng), or Flag-R50A (200 ng), together with EGFP-KDEL (100 ng), for 24 h, followed by immunostaining and confocal microscopy. (**E**) Endo H sensitivity of MHC-I following expression of LSDV003 or the indicated mutants. HEK293T cells (1.5 × 10^6^) were transfected with Flag-LSDV003 or its mutants (400 ng) for 24 h. Cell lysates were treated with Endo H for 2 h before immunoblot analysis. (**F**) Interaction of LSDV003 mutants with endogenous MHC-I. HEK293 cells (1 × 10^7^) were transfected with Flag-LSDV003 or its mutants (2 μg) for 24 h, followed by immunoprecipitation and immunoblotting. (**G**) Effects of R50 substitutions on MHC-I surface expression. HEK293T cells (1 × 10^5^) were transfected with Flag-LSDV003 or the indicated R50 mutants (400 ng) for 24 h, and MHC-I surface expression was analyzed by flow cytometry. (**H**) Interaction of R50 mutants with endogenous MHC-I. HEK293 cells (1 × 10^7^) were transfected with Flag-LSDV003 or the indicated R50 mutants (2 μg) for 24 h, followed by immunoprecipitation and immunoblotting with the indicated antibodies. Data in panels **B** and **G** are presented as mean ± SD (*n* = 3). Statistical significance was determined by one-way ANOVA. **, *P* < 0.01.

Structural modeling predicted two spatially distinct contact regions between LSDV003 and HLA-A, adjacent to the α2 and α3 domains of the HLA-A heavy chain (Fig. 5A). Enlarged views of the predicted interfaces identified several LSDV003 residues positioned near the HLA-A surface, including S148 and K150 at the α2-facing interface and R50, V98, K103, and S199 at the α3-facing interface (Fig. 5A). Several of these residues were positioned in geometries compatible with potential electrostatic or hydrogen-bonding interactions.

To assess the functional relevance of these predicted interface residues, we generated individual point mutants of LSDV003. Flow cytometric analysis showed that, despite being expressed at a level comparable to that of wild-type LSDV003, the R50A mutant largely lost the ability to reduce HLA-I surface expression (Fig. 5B). Confocal microscopy further showed that the R50A mutation did not substantially alter the ER localization of LSDV003 (Fig. 5C), indicating that its loss of function was unlikely to result from gross mislocalization of the viral protein. Consistent with the flow cytometry results, HLA-I remained predominantly localized at the plasma membrane in cells expressing R50A, in contrast to the altered intracellular distribution observed in cells expressing wild-type LSDV003 (Fig. 5D). Endo H digestion assays further showed that HLA-I remained predominantly Endo H resistant in cells expressing R50A, indicating that this mutant failed to efficiently inhibit MHC-I maturation and trafficking through the Golgi apparatus (Fig. 5E).

We next examined whether the functional defect of R50A was associated with impaired interaction with MHC-I. Co-IP assay showed that R50A exhibited markedly reduced association with endogenous HLA-I compared with wild-type LSDV003 (Fig. 5F). To further investigate the contribution of the R50 side chain, we generated R50K and R50H substitutions. Both mutants restored the ability of LSDV003 to reduce MHC-I surface expression to levels comparable to that of the wild-type protein (Fig. 5G). Consistently, R50K and R50H retained association with endogenous HLA-I at levels comparable to wild-type LSDV003 (Fig. 5H). Together, these results indicate that residue R50 contributes to the association of LSDV003 with MHC-I and is required for efficient inhibition of MHC-I trafficking. The restoration of activity by R50K and R50H further suggests that the side-chain properties at residue 50, including side-chain size and charge characteristics, are important for LSDV003 function.

### Comparative analysis of LSDV003 orthologs in MHC-I downregulation

**Fig 6:**
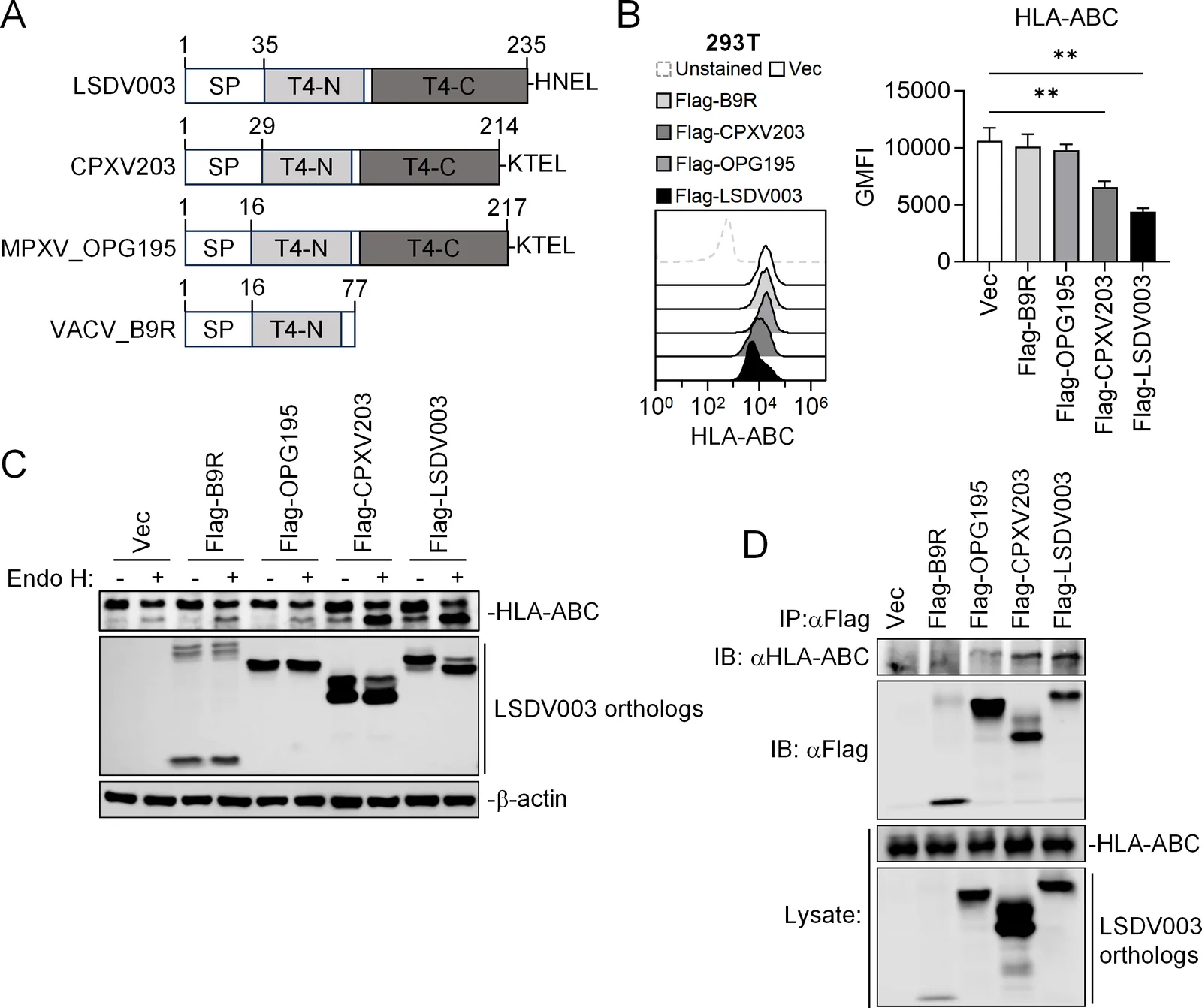
Comparative analysis of LSDV003 orthologs in MHC-I downregulation. (**A**) Domain organization of LSDV003 homologs from CPXV, MPXV, and VACV. (**B**) Effects of LSDV003 homolog overexpression on MHC-I surface expression. HEK293T cells (1 × 10^5^) were transfected with LSDV003 homologs (400 ng) for 24 h. MHC-I surface expression was analyzed by flow cytometry. Data are presented as mean ± SD (*n* = 3). **, *P* < 0.01 (one-way ANOVA). (**C**) Endo H sensitivity of MHC-I following homolog overexpression. HEK293T cells (1.5 × 10^6^) were transfected with LSDV003 homologs (400 ng) for 24 h. Cell lysates were treated with Endo H for 2 h before immunoblot analysis. (**D**) Interaction of homologs with endogenous MHC-I. HEK293T cells (1 × 10^7^) were transfected with the indicated plasmids for 24 h, followed by immunoprecipitation and immunoblot analysis.

We next examined whether other orthopoxviruses use similar mechanisms to downregulate MHC-I expression. BLAST analysis using full-length LSDV003 identified putative homologs in vaccinia virus (VACV) and monkeypox virus (MPXV), namely, B9R (37.2% identity) and OPG195 (29.7% identity), respectively. Sequence analysis revealed that CPXV203 and MPXV_OPG195 contain a putative C-terminal ER retention motif, whereas VACV_B9R lacks both the T4 protein C-terminal domain and any recognizable ER retention motif (Fig. 6A).

To compare their functional activities, we transfected HEK293T cells with each homolog and assessed MHC-I surface expression by flow cytometry and glycan maturation by Endo H digestion. Surprisingly, despite MPXV_OPG195 containing a KTEL motif similar to CPXV203, only CPXV203 and LSDV003 inhibited MHC-I surface expression (Fig. 6B) and blocked cis- to trans-Golgi transport, as suggested by increased Endo H sensitivity (Fig. 6C). We hypothesized that these differences reflect variation in their ability to associate with MHC-I. To test this, we transfected HEK293T cells with each homolog and assessed their interaction with endogenous MHC-I by immunoprecipitation. The results showed that CPXV203 and LSDV003 strongly interacted with HLA-I, whereas OPG195 and B9R exhibited weak or no detectable interaction (Fig. 6D). Taken together, these findings suggest that inhibition of MHC-I trafficking by LSDV003 and its orthologs requires both robust association with MHC-I and a functional C-terminal ER retention motif.

### Deletion of LSDV003 abolishes LSDV-mediated downregulation of MHC-I

**Fig 7:**
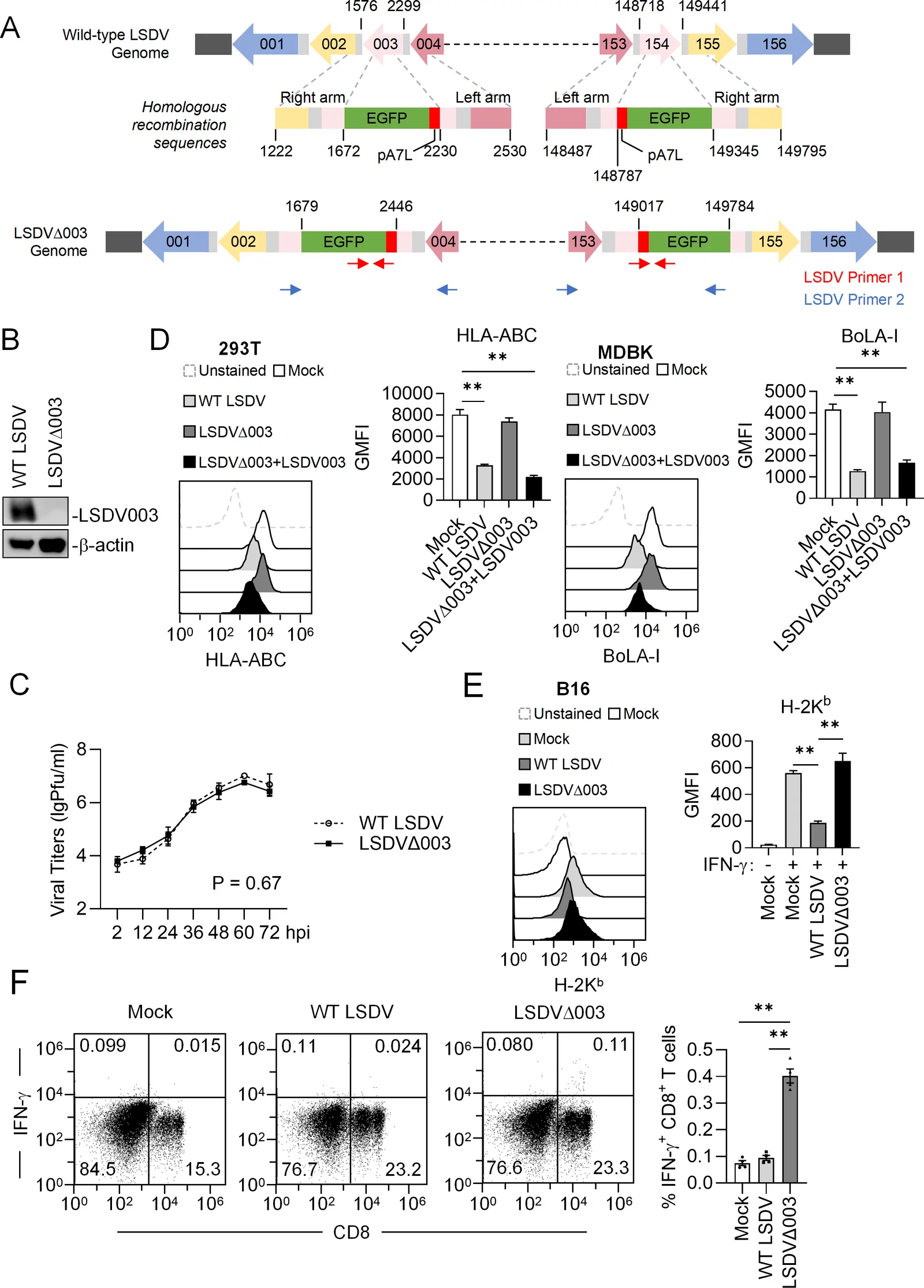
Deletion of LSDV003 abolishes LSDV-mediated MHC-I downregulation. (**A**) Schematic representation of LSDVΔ003 construction. The *LSDV003/154* coding region located within the ITR of the LSDV genome was replaced with EGFP expression cassettes driven by the LSDV pA7L promoter via homologous recombination. Left (LA) and right (RA) homology arms flanking the *LSDV003/154* locus were used to facilitate targeted recombination. The expected genomic organization of the recombinant LSDVΔ003 virus is shown. (**B**) Immunoblot analysis confirming the generation of LSDVΔ003. MDBK cells were infected with WT LSDV or LSDVΔ003 (MOI = 1) for 24 h, followed by immunoblotting with the indicated antibodies. (**C**) Replication kinetics of WT LSDV and LSDVΔ003. MDBK cells were infected at an MOI of 0.01, and viral titers were determined at the indicated time points by plaque assay. (**D**) Effects of LSDV003 deletion on MHC-I surface expression. HEK293T and MDBK cells (1 × 10^5^) were transfected with empty vector or LSDV003 (400 ng) for 24 h and then infected with WT LSDV or LSDVΔ003 (MOI = 1) for an additional 24 h. Surface MHC-I levels were analyzed by flow cytometry. (**E**) Effects of LSDV003 deletion on B16 MHC-I surface expression. B16 cells (5 × 10^5^) were left untreated or pretreated with IFN-γ (40 ng/mL) for 6 h and then infected with WT LSDV or LSDVΔ003 (MOI = 1) for 24 h. MHC-I surface expression was assessed by flow cytometry. (**F**) Effects of LSDV003 deletion on CD8^+^ T-cell activation. Seven-week-old C57BL/6 mice (*n* = 4) were intraperitoneally infected with WT LSDV (2 × 10^6^ PFU). Splenocytes were harvested on day 7 post-infection and co-cultured for 6 h with B16 cells pre-stimulated with IFN-γ for 6 h and infected with WT LSDV or LSDVΔ003. IFN-γ production in CD8^+^ T cells was measured by flow cytometry. Data in panels **C–F** are presented as mean ± SD. Statistical significance was determined by two-way ANOVA (**C**) or one-way ANOVA (**D–F**). **, *P* < 0.01.

To investigate the role of endogenous LSDV003 during infection, we generated a recombinant virus lacking *LSDV003*/*154* (LSDVΔ003) by replacing the *LSDV003*/*154* coding region with an EGFP expression cassette driven by the LSDV A7L promoter (Fig. 7A). Loss of LSDV003 expression was confirmed by immunoblotting analysis of infected cells (Fig. 7B). Multi-step growth curve analysis demonstrated that the replication kinetics of LSDVΔ003 were comparable to those of wild-type LSDV (WT LSDV) (Fig. 7C), indicating that *LSDV003/154* is dispensable for viral replication in MDBK cells. However, infection of HEK293T and MDBK cells revealed that LSDVΔ003 failed to downregulate MHC-I surface expression, whereas WT LSDV efficiently suppressed MHC-I expression (Fig. 7D). Importantly, ectopic expression of LSDV003 in LSDVΔ003-infected cells restored MHC-I downregulation to levels comparable to those observed with WT LSDV (Fig. 7D). These findings demonstrate that LSDV003 is both necessary and sufficient for LSDV-mediated downregulation of MHC-I.

To determine whether LSDV003-mediated MHC-I downregulation affects CD8^+^ T cell activation, C57BL/6 mice, which express the H-2Kᵇ and H-2Dᵇ MHC-I molecules, were infected intraperitoneally with WT LSDV, and splenocytes were harvested at day 7 post-infection. B16 melanoma cells, which share the same H-2^b^ genetic background as C57BL/6 mice, were used as standardized syngeneic target cells to provide a compatible context for CD8^+^ T-cell recognition of infected cells. These cells exhibit low basal MHC-I expression and were therefore pretreated with IFN-γ to upregulate MHC-I expression, followed by infection with WT LSDV or LSDVΔ003 for 24 h. Splenocytes were then co-cultured with the infected B16 cells, followed by intracellular cytokine staining and flow cytometric analysis of IFN-γ production. IFN-γ treatment markedly upregulated H-2K^b^ expression on B16 cells, which was efficiently downregulated by WT LSDV but not by LSDVΔ003 (Fig. 7E). Consistently, stimulation with LSDVΔ003-infected B16 cells resulted in a significantly higher frequency of CD8^+^ IFN-γ^+^ T cells compared with stimulation by WT LSDV-infected cells (Fig. 7F). These results suggest that LSDV003 inhibits MHC-I surface expression, thereby reducing CD8^+^ T-cell effector responses.

## DISCUSSION

In recent decades, LSD has emerged as one of the most significant transboundary animal diseases, posing major economic and food-security concerns. Most currently available LSD vaccines are live attenuated formulations derived from *Capripoxvirus*, including attenuated strains of LSDV, sheeppox virus, or goatpox virus (30). However, these vaccines differ in reactogenicity and efficacy, highlighting the need for improved vaccine strategies. In this study, we focused on characterizing the CMI response during LSDV infection, given its pivotal role in antiviral immunity and vaccine efficacy (31).

Effective CMI requires presentation of viral peptides on MHC-I molecules to CTLs (32). Our results demonstrate that LSDV infection significantly downregulates surface expression of MHC-I in both human and bovine cell lines. Notably, total cellular MHC-I protein abundance was not reduced, indicating that LSDV does not inhibit MHC-I synthesis but instead interferes with intracellular trafficking. Consistent with this interpretation, confocal microscopy and glycosylation assays further revealed that MHC-I trafficking was impaired, with immature MHC-I molecules accumulating in the perinuclear region. This suggests that LSDV blocks MHC-I maturation and export by disrupting its transport from the cis- to trans-Golgi compartments.

Through screening of an LSDV ORF expression library, we identified LSDV003 as the viral protein responsible for MHC-I downregulation. Although a rational homology-based approach using known *Orthopoxvirus* homologs can help prioritize candidate genes, unbiased functional screening enables the identification of viral regulators without relying on prior annotation or predicted functional conservation (33). Several additional proteins, including LSDV054 and LSDV087, also reproducibly reduced MHC-I surface expression, although their effects were less pronounced than that of LSDV003. LSDV054 is annotated as a Fen1-like nuclease, whereas LSDV087 has been reported to possess mRNA-decapping activity and to regulate MITA/STING signaling (34). These functions raise the possibility that their effects on MHC-I surface expression may involve broader alterations in cellular RNA or protein homeostasis. Whether these candidates represent additional MHC-I regulators acting through distinct mechanisms remains to be determined.

LSDV003 contains an N-terminal signal peptide, suggesting entry into the classical ER-Golgi secretory pathway. Its C-terminal HNEL motif resembles known ER retention signals such as KDEL, HDEL, and RDEL (35). Deletion of the HNEL motif leads to increased Golgi localization of LSDV003, supporting the idea that it acts via the ER retention pathway. In mammalian cells, luminal proteins bearing such motifs are recognized by KDEL receptors in the mildly acidic pH of the Golgi, which triggers retrieval back to the ER via COPI-mediated retrograde transport. Although HNEL is less common than canonical retention signals, it is functionally active. For example, the receptor-associated protein in mammalian cells also uses an HNEL signal and binds the KDEL receptor (26). Our findings suggest that LSDV003 exploits this conserved retrieval pathway to retain MHC-I molecules in the ER.

We further demonstrated that LSDV003 interacts with both the MHC-I heavy chain and β2-microglobulin. Structural modeling predicted a ternary HLA-A–β2m–LSDV003 complex in which LSDV003 contacts the lower surface of MHC-I, involving both the HLA-A heavy chain and β2m. LSDV003 harbors a β-sandwich fold, characteristic of the PIE domain family. Despite low sequence conservation, PIE domain-containing proteins share this conserved fold and serve diverse immunomodulatory functions across the *Chordopoxvirinae*, including binding chemokines, inhibiting inflammatory responses, regulating apoptosis, and interfering with MHC-I antigen presentation (28). Mutational analysis identified R50 as an important determinant of LSDV003-mediated MHC-I regulation. Substitution of R50 with alanine markedly reduced the association of LSDV003 with endogenous MHC-I and impaired its ability to inhibit MHC-I trafficking and surface expression, whereas R50K and R50H retained both MHC-I association and inhibitory activity. These findings support an important role for R50 in LSDV003–MHC-I association and functional regulation. Together with the predicted multi-site interface, the mutational data suggest that LSDV003 engages MHC-I through a composite interaction surface rather than through a single dominant contact. The precise molecular basis by which R50 influences the LSDV003–MHC-I interaction remains unclear. R50 may contribute to local interface geometry or participate in a broader network of cooperative contacts rather than acting through a single residue pair, and further structural studies will be required to define the atomic basis of this interaction.

Comparative sequence and domain analyses indicated that LSDV003, CPXV203, VACV_B9R, and MPXV_OPG195 belong to the T4 protein family. The prototypical M-T4 protein from myxoma virus functions as an apoptosis inhibitor; although it contains a C-terminal RDEL motif, deletion of this motif does not disrupt ER localization (36). Despite sequence similarities between OPG195 and the MHC-I inhibitor CPXV203, OPG195 does not downregulate MHC-I surface expression, possibly due to weak or species-specific interactions. VACV_B9R similarly lacks this activity, likely as a consequence of a frameshift mutation that generates a truncated protein (37). These evolutionary divergences may help explain why VACV elicits strong memory CD8^+^ T-cell responses and serves as a robust vaccine vector.

To define the role of LSDV003 in the context of authentic LSDV infection, we generated an LSDV003-deficient virus. This mutant retained normal replication capacity but lost the ability to downregulate cell surface MHC-I expression, demonstrating that LSDV003 is a major determinant of LSDV-mediated MHC-I suppression. In contrast, CPXV employs at least two proteins, CPXV012 and CPXV203, to inhibit MHC-I function, such that deletion of either protein only partially restores MHC-I surface expression (20). This difference highlights a streamlined immune evasion strategy employed by LSDV.

In conclusion, our study identifies LSDV003 as a key immune evasion protein that enables LSDV to suppress cellular immunity by downregulating MHC-I surface expression. Elucidation of this mechanism advances our understanding of LSDV pathogenesis and provides important insights for the rational design of live attenuated vaccines and antiviral strategies targeting immune evasion pathways.

## MATERIALS AND METHODS

### Cells, virus, and reagents

HEK293T, HeLa, and MDBK cells (ATCC) were maintained in DMEM (C11965500BT, Gibco) supplemented with 10% FBS (SA211.02, Cellmax), 100 U/mL penicillin, and 100 U/mL streptomycin (15140163, Gibco). B16 and MC38 cells were previously described (38).

The LSDV strain (LSDV/China/Hainan/2021) was described previously (39). The LSDV-EGFP reporter virus was generated by insertion of EGFP into the TK locus, which is widely used for insertion of heterologous genes in recombinant poxviruses (40, 41). The LSDVΔ003 virus was generated by replacement of the LSDV003 and LSDV154 ORFs with EGFP expression cassettes through homologous recombination.

Reagents included TrypLE Express (12604021, ThermoFisher Scientific); EDTA (10 mM, pH 8.0; BL518A, Biosharp); Flow Cytometry Perm Buffer (PF00017, Proteintech); RNAiso Plus (#9109, TaKaRa); HiScript III RT SuperMix (R323, Vazyme); SYBR Green Supermix (Q312, Vazyme); Lipofectamine 3000 (L3000015, Invitrogen); FuGENE HD (E2311, Promega); EZ-Link Sulfo-NHS-SS-Biotin (A39258, Thermo); streptavidin agarose (P2159, Beyotime); endoglycosidase H (P0702S, NEB); protein A+G agarose (P2055, Beyotime); and the inhibitors brefeldin A, aprotinin, leupeptin, β-glycerophosphate, and sodium orthovanadate (MCE).

Antibodies used were anti-HA (H6908, Sigma); anti-Flag (F3165, Sigma); HRP-Flag (ZB15939, Servicebio); mouse and rabbit IgG controls (I5381, I5006, Sigma); anti-mouse IgG (H+L), Alexa Fluor 594 (8890, CST); anti-rabbit IgG (H+L), AF647 (A0468, Beyotime); DAPI (S36920, Thermo); APC-conjugated HLA-ABC (17-9983-42), CD4 (17-0042-82), and CD8a (17-0081-82), FITC-conjugated HLA-ABC (11-9983-42), CD71 (11-0719-42), CD3e (11-0031-82), and PE-conjugated IFN-γ (12-7311-82) (all from eBioscience); FITC-conjugated CD46 (MA1-19601, Invitrogen); HLA class I ABC (15240-1-AP, Proteintech); GFP (66002-1-Ig, Proteintech); ATP1A1 (14418-1-AP, Proteintech); CD28 (16-0281-82, eBioscience); GAPDH (60004-1-Ig, Proteintech); and β-actin (sc-47778, Santa Cruz Biotechnology).

### Constructs

Coding sequences of LSDV003 and its orthologs were synthesized by GenScript (Nanjing, China). Flag-tagged LSDV003, HA-tagged HLA-A, HLA-B, HLA-C, and β₂-microglobulin (β₂m) expression plasmids were generated using standard molecular cloning strategies. LSDV003 point mutants were created by site-directed mutagenesis using overlap extension PCR.

### Transfection

HEK293T cells were transfected using the standard calcium phosphate precipitation method. MDBK and HeLa cells were transfected with Lipofectamine 3000 or FuGENE HD reagent according to the manufacturer’s instructions.

### Flow cytometry

Cells were harvested by either TrypLE Express Enzyme or PBS containing 10 mM EDTA and washed twice with FACS buffer (PBS, 1% FBS). For surface staining, cells were incubated at 4°C for 1 h with indicated antibodies. After two washes with FACS buffer, cells were fixed in 4% paraformaldehyde for 30 min at 4°C in the dark. Finally, samples were washed and resuspended in FACS buffer for analysis on a flow cytometer. Data were processed using FlowJo software.

### Reverse transcription-quantitative polymerase chain reaction

Total RNA was isolated from cells using RNAiso Plus. The RNA was reverse transcribed with HiScript III RT SuperMix according to the manufacturer’s protocol. Relative expression was calculated using the 2^−ΔΔCt^ method with *GAPDH* as the reference gene. The primer sequences are as follows: human HLA-ABC, forward 5′-GCCTACGACGGCAAGGATTA-3′ and reverse 5′-TCCTTCCCGTTCTCCAGGTA-3′; human GAPDH, forward 5′-GACAAGCTTCCCGTTCTCAG-3′ and reverse 5′-GAGTCAACGGATTTGGTCGT-3′; bovine BOLA, forward 5′-TCACCCTGAGATGGGAACCT-3′ and reverse 5′-TGGGCACTGTCACTGCTTG-3′; and bovine GAPDH, forward 5′-AGGTCGGAGTGAACGGATTC-3′ and reverse 5′-ATGGCGACGATGTCCACTTT-3′.

### Cell surface protein biotinylation and isolation

Cells were washed three times with ice-cold PBS (pH 8.0) and incubated on ice for 30 min with 1 mg/mL freshly prepared sulfo-NHS-SS-biotin in PBS. Excess biotin was quenched with 100 mM glycine in PBS for 10 min at 4°C, followed by two PBS washes. Cells were lysed by sonication in lysis buffer (50 mM Tris-HCl, pH 7.5; 150 mM NaCl; supplemented with a protease inhibitor cocktail). Lysates were clarified by centrifugation at 12,000 × *g* for 10 min at 4°C, and the supernatant was collected. Biotinylated proteins were enriched with streptavidin-agarose beads overnight at 4°C and washed with lysis buffer. Bound proteins were eluted by boiling in SDS sample buffer and analyzed by SDS-PAGE, followed by western blotting with the indicated antibodies.

### Confocal microscopy

Cells grown on glass coverslips were fixed in 4% PFA for 30 min at room temperature, permeabilized with 0.2% Triton X-100 in PBS for 30 min, and blocked with 5% BSA in PBS for 1 h. Samples were incubated overnight at 4°C with the primary antibodies diluted in blocking buffer, washed three times with PBS, and then incubated for 1 h at room temperature in the dark with fluorophore-conjugated secondary antibodies. After washing, coverslips were mounted in antifade medium containing DAPI. Images were acquired on a Leica Stellaris 5 confocal microscope under identical acquisition settings and processed using Leica LAS X software.

### Endo H digestion assay

Cells were lysed in 1% NP-40 and centrifuged at 12,000 × *g* for 5 min at 4°C. Supernatants were denatured in 1× glycoprotein denaturing buffer at 95°C for 10 min, cooled to room temperature, and incubated with or without 1,000 U of Endo H in 1× GlycoBuffer 3 at 37°C for 2 h. Reactions were terminated by adding SDS-PAGE loading buffer, and samples were analyzed by SDS-PAGE, followed by western blotting with the indicated antibodies.

### Co-immunoprecipitation

Cells were lysed on ice for 10 min in IP lysis buffer (50 mM Tris-HCl, pH 7.4; 1% NP-40; 150 mM NaCl; 1 mM EDTA; protease inhibitors), followed by brief sonication and centrifugation at 14,000 rpm for 15 min at 4°C. Supernatants were incubated with the indicated antibodies and protein A/G agarose beads for 4 h at 4°C with gentle rotation. Beads were washed three times with high-salt buffer (50 mM Tris-HCl, pH 7.4; 650 mM NaCl), and bound proteins were eluted by boiling in SDS-PAGE sample buffer. Samples were analyzed by SDS-PAGE and immunoblotting.

### Complex structure prediction

The HLA-A–β₂m–LSDV003 complex was modeled using AlphaFold Server (29). Sequences corresponding to LSDV003 residues 31–216, HLA-A residues 25–305, and β₂m residues 21–119 were submitted as three separate protein chains using the default settings. The top-ranked model was selected for structural visualization and interface analysis. Prediction confidence was assessed using the per-residue pLDDT, PAE, and pairwise ipTM scores provided by the server.

### Purification of LSDV003 and generation of polyclonal antibody

The coding sequence of LSDV003 was cloned into pET-30c vector with an N-terminal 6xHis tag and transformed into *Escherichia coli* BL21 cells. Protein expression was induced with 0.5 mM IPTG for 10 h at 37°C. Cells were harvested, lysed by sonication, and clarified by centrifugation. The His-tagged LSDV003 was purified under native conditions using Ni-NTA agarose, eluted with imidazole, and dialyzed against PBS. Protein purity and yield were confirmed by SDS-PAGE with Coomassie Brilliant Blue staining.

For antibody production, 100 μg of purified LSDV003 was emulsified with an equal volume of Freund’s complete adjuvant and injected subcutaneously into 8-week-old BALB/c mice. Boosters were administered every 2 weeks. After the fourth immunization, sera were collected and stored at −80°C.

### Generation of recombinant virus

LSDVΔ003 was generated by replacing both duplicated copies, *LSDV003* and *LSDV154*, with EGFP expression cassettes. The recombinant plasmid (pUC18-LA-EGFP-RA) was constructed by inserting the EGFP reporter gene, driven by the LSDV pA7L promoter, between 300-bp left and 450-bp right homology arms (LA and RA) flanking the *LSDV003*/*LSDV154* gene, into the pUC18 backbone. MDBK cells were infected with wild-type LSDV for 2 h and subsequently transfected with the recombinant plasmid. After 48 h of culture, cells were harvested, and recombinant virus was purified through multiple rounds of limiting dilution. The purity of LSDVΔ003 was confirmed by PCR and immunoblotting.

To validate the purity and distinguish between wild-type and recombinant viruses, specific primers were designed as follows: LSDV Primer 1, forward 5′-ACCTGGAGGACTTCTAACAG-3′ and reverse 5′-TCTCAACGTATGATTTCATTTCTG-3′; LSDV Primer 2, forward 5′-CATTTTTACTAGTAGTGTAAGCGG-3′ and reverse 5′-AAATTTACCAACACTGCACG-3′.

LSDV Primer 1 is used to verify the purity of the virus and can amplify wild-type LSDV (254 bp) but not recombinant virus. LSDV Primer 2 is used to verify the insertion sequence and amplify wild-type LSDV (613 bp) and LSDVΔ003 (836 bp).

### Viral replication kinetics

MDBK cells were seeded in 12-well plates and grown to approximately 90% confluence. Cells were infected with LSDV at an MOI of 0.01. After 1 h of viral adsorption at 37°C, the inoculum was removed, and the cells were washed three times with PBS. Fresh maintenance medium was then added, and the infected cells were incubated at 37°C in a humidified atmosphere containing 5% CO_2_ for the indicated time points. At each time point, both cells and culture supernatants were collected and subjected to three freeze-thaw cycles to release intracellular virus. The samples were then centrifuged at 12,000 rpm for 5 min, and the clarified supernatants containing virus were subsequently used for determination of viral titers by plaque assay.

### Isolation of splenocytes

Spleens were aseptically harvested from mice and placed into cold RPMI 1640 medium supplemented with 2% FBS. Splenocytes were obtained by gently pressing the spleens through a 70 μm cell strainer using the plunger of a syringe. The single-cell suspension was centrifuged at 300 × *g* for 5 min at 4°C, and red blood cells were lysed using 1× RBC lysis buffer for 2–3 min at room temperature, followed by washing with RPMI 1640. Cells were counted and resuspended in serum-free freezing medium and stored at −80°C before use.

### Intracellular cytokine staining

Splenocytes (2 × 10^6^ cells) were co-cultured with infected B16 cells (1 × 10^6^ cells) for 1 h in restimulation medium consisting of RPMI 1640 supplemented with 10% FBS and 1 μg/mL anti-CD28 antibody. Brefeldin A was then added to a final concentration of 5 μg/mL, followed by an additional 5 h of incubation. Cells were harvested and stained with fluorochrome-conjugated monoclonal antibodies against CD3 and CD8 for 30 min at 4°C in the dark. After surface staining, cells were washed twice with FACS buffer, fixed with 4% paraformaldehyde, and permeabilized using flow cytometry permeabilization buffer. Intracellular staining was then performed using an anti-IFN-γ antibody. After two additional washes with FACS buffer, samples were analyzed by flow cytometry.

### Biosafety statement

All experiments involving live LSDV were conducted in enhanced biosafety level 3 (P3) facilities at the Lanzhou Veterinary Research Institute, Chinese Academy of Agricultural Sciences, with approval from the Ministry of Agriculture and Rural Affairs (approval number: 07140020250302) and the China National Accreditation Service for Conformity Assessment (approval number: CNAS BL0098).

### Statistical analysis

Unless otherwise indicated, quantitative data are presented as mean ± SD from at least three independent experiments. Statistical analyses were performed using unpaired Student’s *t*-test, one-way ANOVA, or two-way ANOVA as appropriate. Statistical significance was defined as *P* < 0.05 (*) or *P* < 0.01 (**).

## Data Availability

The data supporting the findings of this study are available from the corresponding author upon reasonable request. Structural models generated in this study were obtained using AlphaFold Server and are described within the article. All other data generated or analyzed during this study are included in the article.
